# Seven-Month Vitamin D Deficiency Inhibits Gastric Epithelial Cell Proliferation, Stimulates Acid Secretion, and Differentially Alters Cell Lineages in the Gastric Glands

**DOI:** 10.3390/nu15214648

**Published:** 2023-11-02

**Authors:** Shaima Sirajudeen, Iltaf Shah, Sherif M. Karam, Asma Al Menhali

**Affiliations:** 1 Department of Biology, College of Science, United Arab Emirates University (UAEU), Al Ain 15551, United Arab Emirates; 201890080@uaeu.ac.ae; 2 Zayed bin Sultan Al Nahyan Center for Health Sciences, United Arab Emirates University (UAEU), Al Ain 15551, United Arab Emirates; altafshah@uaeu.ac.ae (I.S.); skaram@uaeu.ac.ae (S.M.K.); 3 Department of Chemistry, College of Science, United Arab Emirates University (UAEU), Al Ain 15551, United Arab Emirates; 4 Department of Anatomy, College of Medicine and Health Sciences, United Arab Emirates University (UAEU), Al Ain 15551, United Arab Emirates

**Keywords:** Vitamin D, gastric acid, apoptosis, proliferation, parietal cells, zymogenic cells

## Abstract

Vitamin D (VD) deficiency can result from insufficiency of either light exposure or VD intake. We investigated the biological effects of VD deficiency for 7 months on the mouse gastric glands. Varying degrees of VD deficiency were induced in C57BL/6 mice by keeping them on standard diet with constant-dark conditions (SDD) or VD deficient diet with constant-dark conditions (VDD). Samples of serum, glandular stomach, and gastric contents were collected for LCMS/MS, RT-PCR, immunohistochemistry, and acid content measurements. Both SDD and VDD mice had a significant decline in 25OHVD metabolite, gastric epithelial cell proliferation, and mucin 6 gene expression. These effects were enhanced with the severity of VD deficiency from SDD to VDD. Besides and compared to the control group, SDD mice only displayed a significant increase in the number of zymogenic cells (*p* ≤ 0.0001) and high expression of the adiponectin (*p* ≤ 0.05), gastrin (*p* ≤ 0.0001), mucin 5AC (*** *p* ≤ 0.001) and the Cyclin-dependent kinase inhibitor 1A (**** *p* ≤ 0.0001). These phenotypes were unique to SDD gastric samples and not seen in the VDD or control groups. This study suggests that the body reacts differently to diverse VD deficiency sources, light or diet.

## 1. Introduction

Vitamin D (VD) insufficiency affects approximately 50% of the population, and VD deficiency affects around one billion individuals globally [1]. Calcium homeostasis, bone metabolism, and several other biological functions are regulated by VD [2,3]. In humans, it has been estimated that 50 to 90 percent of VD is formed in the skin when exposed to sunlight, with the remainder coming from the diet [4]. However, cutaneous VD production diminishes with age [5]. Although VD has been extensively studied and its significance in several organs has been confirmed, little is known about its molecular and cellular effects that regulate the growth and homeostasis of the gastric glands [6].

The metabolite of VD, 25-hydroxy VD (25OHD), is a potential biomarker for clinical screening. However, other metabolites, such as epimers, play important roles in determining VD levels in the body [7]. Epimers are VD metabolites that have crucial functions throughout intrauterine life and during postnatal development [8]. Previous research indicated that mice kept in the dark for a period of 12 months exhibited elevated levels of VD epimers in their serum, as determined through liquid chromatography–tandem mass spectrometry (LC–MS/MS) analysis [9]. LC–MS/MS analysis is advantageous because it concurrently analyzes active and inactive metabolites of VD, is extremely accurate and aids in the discovery of metabolites that have not been extensively researched [8]. Serum samples are typically used for the LC–MS/MS analysis of VD metabolites [10].

The gastric glands are populated by different types of cells, including surface mucous, mucous neck, parietal, zymogenic, enteroendocrine (including G, D, and enterochromaffin-like or ECL), and tuft cells [11,12]. In mice and humans, these cells arise from multipotent stem cells via immature committed progenitor cells in the glandular isthmus region. Therefore, gastric stem/progenitor cells are responsible for the self-renewal and homeostasis of the gastric epithelium [11,12]. To differentiate into various specialized cells, progenitor cells move in two directions: upward into the pit region and downward toward the base of the gland [13,14]. This occurs with control of signaling pathways, such as Notch [15], Wnt [16], hedgehog [17], and runt-related transcription factor 3 [18,19]. Environmental, cellular, hormonal, growth, and neurological factors are also involved [20]. The disruption of these components may have a deleterious effect on gastric homeostasis, leading to disorders such as gastric cancer [21]. However, the mechanism by which this occurs remains unknown and requires further study.

Acid production by parietal cells is also controlled by several factors including parasympathetic nerve fibers and hormones, such as gastrin, histamine, ghrelin, glucagon-like peptide 1, and somatostatin. Variations in these factors may disturb normal glandular physiology and morphology [22]. Acetylcholine, a mediator of the vagal pathway, acts on parietal cells via the M3 receptor in response to stimuli such as thought, sight, and smell of food. Upon food intake, G cells are stimulated resulting in gastrin release. Gastrin secretion stimulates parietal cells by binding to gastrin/CCK-B receptors on parietal cells and ECL cells [23]. Gastrin increases ECL cell histamine production, which increases acid secretion by binding to H2 receptors. D cells in the antrum also monitor stomach contents and pH. When the luminal pH drops below 3.5, D cells produce somatostatin, which inhibits G cell activity [24]. Somatostatin, produced by D cells, inhibits both parietal and ECL cells [25].

In a recent study, the effects of long-term (12-month) VD deficiency on gastric homeostasis revealed major alterations in the gastric glands [9]. Hence, in the current study, we aimed to investigate the effects of mild and severe VD deficiency on the gastric glands by complete dark exposure alone or with a VD-deficient diet for 7 months, to reveal the cell biological features of the transitional phase toward long-term VD deficiency.

## 2. Materials and Methods

### 2.1. Experimental Animals: Mice and Diets

This study was approved by the Animal Research Ethics Committee of United Arab Emirates University (Protocol # ERA-2017_5684). Three-week-old C57BL/6J mice were acquired from the Animal Facility of the College of Medicine and Health Sciences at United Arab Emirates University. The mice were separated according to sex and maintained in a specific pathogen-free environment. The mice were housed in autoclaved cages with filter tops at a temperature of 23 ± 1 °C and relative humidity of 50% ± 10%, and they were handled in a laminar air flow hood. The mice (*n* = 36) were segregated into three groups, namely standard diet/light (SDL), standard diet/dark (SDD), and VD deficient/dark (VDD) for 7 months (Figure S1), according to VD diet and ultraviolet (UV) light conditions. The UV lamps were provided by Bio-Medical Scientific Services (BIOMSS) LLC, Abu Dhabi, United Arab Emirates. The mice were irradiated using Philips Xitanium LED Driver 44 W lamp (Philips, Amsterdam, Hungary) emitting broadband LED, 275–900 nm, and delivered 2.9 kJ/m^2^ of irradiance onto mice. The lamp was positioned at 25 to 30 cm above the mice [26]. The control group of mice, Standard Diet Light (SDL) was maintained on a standard AIN-93G rodent diet, which contained 1000 IU/kg VD3 (D10012Gi, Research Diet, New Brunswick, NJ, USA), with cycles of 12 h UV light and 12 h dark. The second group called the Standard Diet Dark (SDD) group was fed the standard diet and maintained in the dark with no overhead UV light. The third VD deficient Dark (VDD) group was maintained in the dark and fed upon a specially formulated VD-deficient diet with a VD level of 25 IU/kg, D17053003 (Research Diet, New Brunswick, NJ, USA) [9]. The objective was to create mild and severe VD deficiency in the SDD and VDD groups of animals respectively.

### 2.2. Tissue Processing for Microscopic Analysis Using Lectin-Histochemistry and Immunohistochemistry

Anterior portion of the gastric corpus tissues were fixed in Bouin’s fixative overnight. Paraffin sections (4 μm) were prepared for microscopic examination using different stains, antibodies, and lectins to study general morphology, different gastric cell markers, proliferation, and apoptosis in the corpus.

A hematoxylin and eosin (H&E) staining kit (ab245880, Abcam, Cambridge, UK) was used for general histological evaluation as previously described [27]. The sections were deparaffinized, rehydrated, and incubated with H&E for 5 min and 3 min, respectively.

Alcian blue–periodic acid Schiff (AB–PAS) (Thermo Scientific™ Richard–Allan Scientific™ Chromaview™, Thermo Fisher Scientific, Singapore Science Park 2, Singapore 117528) method was used to identify mucous cells. The sections were stained with the Alcian blue pH 2.5 stain solution for 30 min, periodic acid solution for 5 min, and Schiff reagent for 15 min. Nuclear staining was performed by immersing the sections in hematoxylin and finally mounting them in dibutylphthalate polystyrene xylene (DPX).

*Ulex europeus* agglutinin I (UEA-I) conjugated to Rhodamine (1 in 1000; RL-1062-2, Vector Laboratories, San Francisco, CA, USA) and *Griffonia simplicifolia* II (GS-II) lectin conjugated to Alexa Flour™ 488 (1 in 1000; L21415, Invitrogen, Thermo Fisher Scientific, Life Technologies, Carlsbad, CA, USA) were used to label surface mucous cells and mucous neck cells, respectively. Parietal cells were identified using an anti-proton pump (H,K-ATPase β subunit) mouse monoclonal antibody (1 in 5000; D032-3, Medical and Biological Laboratories, Tokyo, Japan). The sections were rapidly treated with a blocking reagent (1% bovine serum albumin [BSA]) for 1 h, followed by overnight incubation at 4 °C with primary antibodies. After washing, the sections were incubated for 1 h at room temperature with biotin-SP-conjugated goat anti-rat IgG (H + L) (1:1000; 112-065-003, Jackson ImmunoResearch, West Grove, PA, USA) and goat anti-rabbit IgG H&L (TRITC) (1 200; ab6718, Abcam, Cambridge, UK) antibodies. The sections were counterstained with hematoxylin and mounted with DPX.

VD receptor (VDR) and protein disulfide-isomerase A3 (PDIA3) were identified using the VD3 receptor antibody (D2K6W) rabbit mAb (1 in 12,000; Cell Signaling Technology, Danvers, MA, USA) and the ERp57 antibody (4E69, 1:200; sc-71075; Santa Cruz Biotechnology, Santa Cruz, CA, USA), respectively. Antigen retrieval for co-immunofluorescence staining was performed at 96 °C for 10 min using a citrate buffer (pH–6.0). The tissue sections were blocked with 1% BSA and incubated at room temperature with primary antibodies against Erp57 and VDR for 1 h. The sections were then washed with 1X phosphate-buffered saline and incubated with Cy3- or Alexa Fluor 448-conjugated goat anti-rat secondary antibodies (1:100; 112-165-003 or 112-545-003; Jackson ImmunoResearch) for 1 h at room temperature. DAPI (ab104139; Abcam, Cambridge, UK) was used to stain the nuclei and as a mounting agent. Primary antibodies were absent in the negative controls.

To identify proliferating cells, tissue sections of all mice were processed for incubation with PC10 mouse mAb (2586, 1 in 12,000; Cell Signaling Technology, Danvers, MA, USA) which is specific for proliferating cell nuclear antigen (PCNA). The secondary antibody was a biotinylated anti-mouse antibody (1 in 50; Invitrogen, Thermo Fisher Scientific, Life Technologies, Carlsbad, CA, USA).

The CellSens software (CellSens 1.9.11514.0) and Olympus IX83 microscope (Tokyo, Japan) was used for the imaging and quantification of ZCs and cells positive for H,K-ATPase and PCNA, in addition to measuring gland height from the luminal surface of the stomach to the gland bottom. Data are presented as the number of positive cells per field.

### 2.3. Gene Expression Analyses

#### 2.3.1. RNA Extraction

The TRIzol reagent (Invitrogen, Thermo Fisher Scientific, Waltham, MA, USA) was used to extract total RNA from the anterior and posterior walls of the mouse stomach corpus. Rnase-free Dnase Kit (Qiagen, Hilden, Germany) was used for RNA purification. To isolate RNA, 50–100 mg of stomach tissue was homogenized in the TRIzol reagent and incubated at room temperature for 5 min. Tissue lysates were treated with chloroform for 3 min and centrifuged at 12,000× *g* at 4 °C for 15 min. 0.5 mL of isopropanol was added, and the aqueous phase was centrifuged at 4 °C for 10 min at ≥12,000× *g*. The total RNA precipitate was resuspended in 75% ethanol. The total RNA was precipitated as a white gel and resuspended in 75% ethanol. The pellet was washed, air-dried, incubated at 55–60 °C for 10–15 min, and resuspended in nuclease-free water. The RNA samples were transferred to a spin column and treated with the Rnase-free Dnase kit and incubated for 15 min. The RNA was eluted from the column in nuclease-free water after several washing steps. Before using it for downstream processing or storing at −80 °C, the quality of RNA samples was confirmed using Thermo Scientific NanoDrop 2000 Spectrophotometer and agarose gel electrophoresis.

#### 2.3.2. Complementary DNA (cDNA) Synthesis

One microgram of RNA was used for cDNA synthesis using the iScript cDNA synthesis kit (Bio-Rad, Hercules, CA, USA).

#### 2.3.3. Quantitative Reverse Transcription Polymerase Chain Reaction (qRT-PCR)

qRT-PCR was performed to analyze the expression of genes specific to adiponectin [10], gastric epithelial cell lineages, VD receptors, PDIA3 and VDR, PDIA3/VDR direct target genes [10], and stem cell markers [28]. cDNA samples in triplicates were used for qRT-PCR reactions using PowerUp SYBR Green Master Mix (A25742, Applied Biosystems, Thermo Fisher Scientific, Carlsbad, CA, USA) in a QuantiStudio^®^ 5 Real-Time PCR equipment (Applied Biosystems, Thermo Fisher Scientific, Foster City, CA, USA). All primers used are listed in Supporting Information, Table S1. To determine relative fold change in gene expression, the delta–delta cycle threshold (DDCt) method was used. The expression of glyceraldehyde-3-phosphate dehydrogenase (Gapdh) gene was used to normalize changes in the expression of the other specific genes.

### 2.4. Measurement of Serum VD Levels by LC–MS/MS

LC-MS/MS analysis has been performed to measure the levels of different VD metabolites in the serum of animals [19,29]. Serum was separated from 200 μL–1 mL of blood by centrifuging at 12,000× *g* for 15 min at 4 °C. The serum was stored at −80 °C until further analysis. The method was validated by Food and Drug Administration and US requirements [30].

### 2.5. Gastric Acid Content Measurement

Stomach tissues were obtained from the three groups of mice. The samples were dissected along the greater curvature, cleaned with a 0.9% sodium chloride solution (pH 7.0), and centrifuged at 1848× *g* (5000 rpm) for 10 min. The acidity of the samples was measured by titration with 0.005 mol/L NaOH [31,32]. The acid equivalent per kilogram of body weight (μEq/kg) was calculated by normalizing the results to the body weight of the mice.

### 2.6. Statistical Analysis

Statistical analyses were performed using GraphPad Prism 7.03 (San Diego, CA, USA). Most qRT-PCR data met the criteria of D’Agostino and Pearson normality tests. The data for qPCR, morphometric analysis, and VD metabolites were expressed as the means ± standard error (SE). The analysis was performed using either one- or two-way analysis of variance (ANOVA) followed by Dunnett’s multiple comparison posthoc test.

## 3. Results

### 3.1. Mild to Severe VD Deficiency Induced Changes in Body Weights

**Figure 1:**
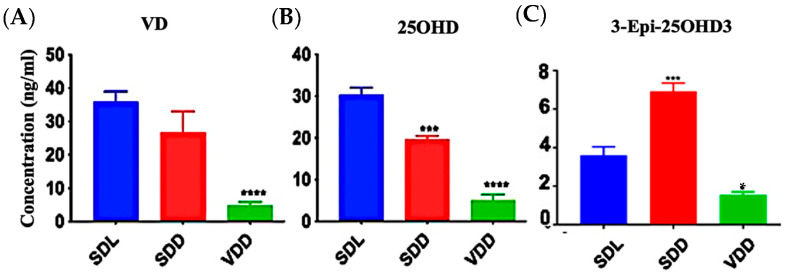
Effect of Light and Diet on VD Metabolites. The sera of all mice (*n* = 8–11 per group) were prepared to detect the levels of VD metabolites: (**A**) 25-hydroxy VD, (**B**) VD, and (**C**) 3-Epi-25OHD3 in ng/mL by using LC-MS/MS. The data are presented as the mean ± SE. The one-way analysis of variance was performed for data analysis. The significance of differences was determined as compared with the control group: * indicates significant differences from the control group. * *p* < 0.05, *** *p* ≤ 0.001, **** *p* ≤ 0.0001.

Significant VD deficiency/insufficiency occurred over the course of seven months. Figure 1A–C shows that diets containing 25 IU/kg VD3 substantially decreased serum 25OHD and VD levels. In comparison to the SDL (3.6 ± 1.0 ng/mL) and VDD (1.6 ± 0.2 ng/mL) groups, 3-epi-25OHD3 levels were higher in the SDD group (6.9 ± 1.0 ng/mL) of mice. The various VD metabolites (ng/mL) as detected by LC-MS/MS analysis in the serum of the three groups of 7M mice including VD3, VD2, 25OHD2, and 25OHD3, were also decreased dramatically (Figure S2A–D, Table S1). VD levels in the hair samples of the VDD mice were not detectable (Figure S2E).

**Figure 2:**
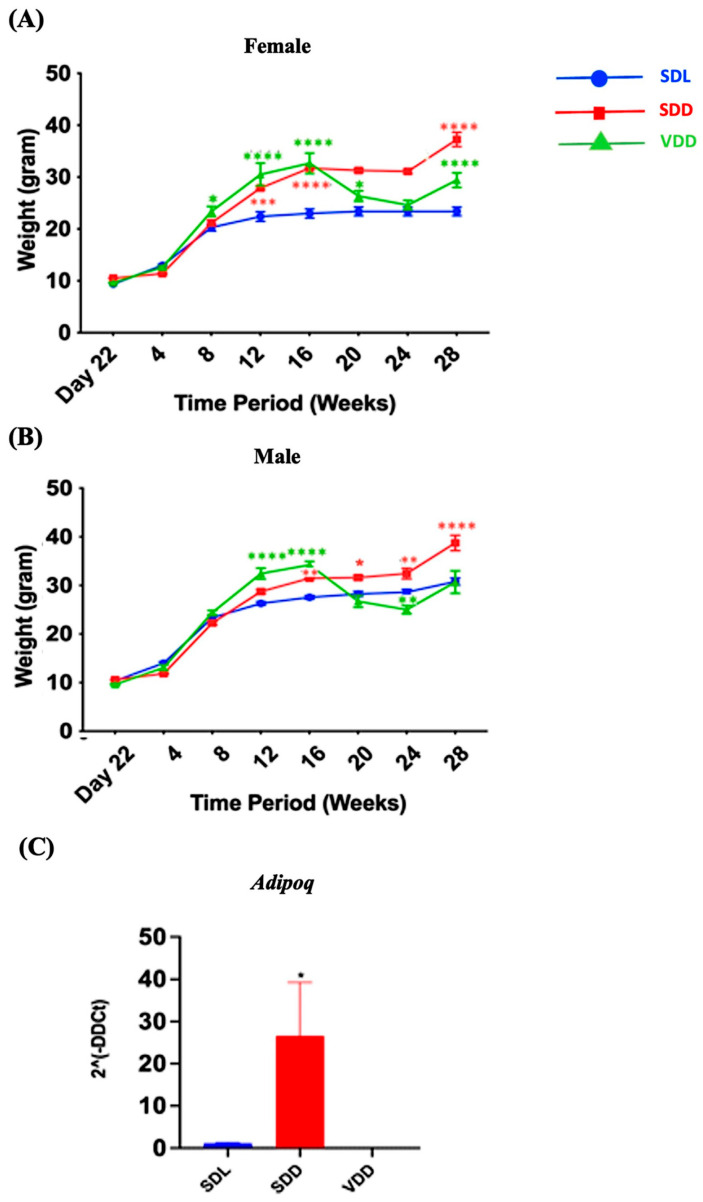
Variations in the Bodyweights of Mice over the Experimental Period. Three-week-old mice were separated based on sex (**A**) female and (**B**) male and grouped into Standard Diet Light (SDL), Standard Diet Dark (SDD), and VD deficient Dark (VDD) groups (*n* = 8–9 per group). The mice were observed for up to 28 weeks, beginning on day 22 (week 3). The body weight of each mouse was measured weekly, and group averages were calculated. (**C**) Gene expression (2ˆ(-DDCt)) of the fat marker *Adipoq*. Data are presented as the mean ± SE. The one-way analysis of variance was performed for data analysis. * indicates significant differences from the control group. * *p* < 0.05, ** *p* < 0.01, *** *p* < 0.001, **** *p* < 0.0001.

There was a remarkable trend in weight change over time. The body weights of the animals in each of the three groups varied (Figure 2A,B). For instance, a considerable gain in body weight was observed in the SDD group, which was maintained in the dark and were on a regular diet. Both genders exhibited the same pattern. In SDD and VDD mice, a considerable weight gain was observed from 8 to 16 weeks. However, body weight gain in the VDD group ceased after 16 weeks. In the SDD group, there was an increase in *Adipoq* (*p* < 0.05) (Figure 2C) gene expression level. Presently, we do not have a conclusive explanation for this phenomenon. However, we believe that factors like gender, age, endocrine dynamics, or epigenetic modifications within the murine cohort, may have exerted an influence on the observed variations in body weight among the SDD and VDD groups. Further investigations are imperative to confirm these findings.

### 3.2. Increase in ZCs and Immune Cell Infiltration in the Submucosa of SDD Mice

**Figure 3:**
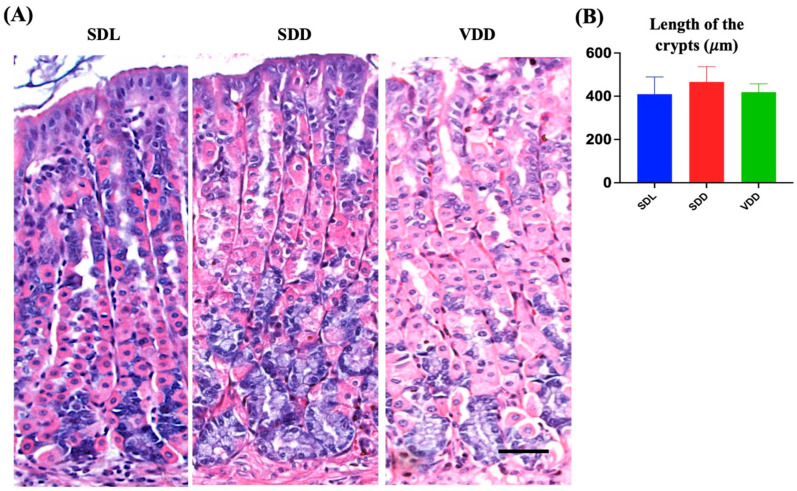
Variations in the Architecture of the Stomach Gland in Seven-month-old Vitamin D (VD)-deficient Mice. (**A**) H&E staining was performed to examine the morphology of the mouse gastric corpus. (**B**) The average lengths (μm) of the stomach glands measured in light micrographs do not show significant differences in the three groups of mice (*n* = 8–9 per group). Data are presented as the mean ± SE and analyzed by the one-way analysis of variance.

We examined the effect of VD deficiency resulting from darkness and diet on the stomach epithelium. The histological examination of mouse gastric tissue sections revealed that the SDL mice had long, closely packed corpus gastric glands. However, the gastric glands of the SDD group contained more basophilic ZCs than those of the SDL and VDD groups. In comparison, the neck regions of the gastric glands of VDD mice in the SDL group showed noticeable dilatation (Figure 3A). The stomachs of SDD and VDD mice exhibited immune cell infiltration, with a higher rate in SDD mice (Figure 3B).

Based on these findings, a thorough examination of the mouse gastric mucosa was performed to study its phenotype. The gastric mucosae and gastric gland heights were normal (Figure 3B, and there were significant changes in the different cellular lineages of the SDL, SDD, and VDD groups, which was revealed by microscopic examination as shown below. No gender-specific histological changes were observed.

### 3.3. Intermediate VD Deficiency Increased ZC Lineage in SDD Mice

**Figure 4:**
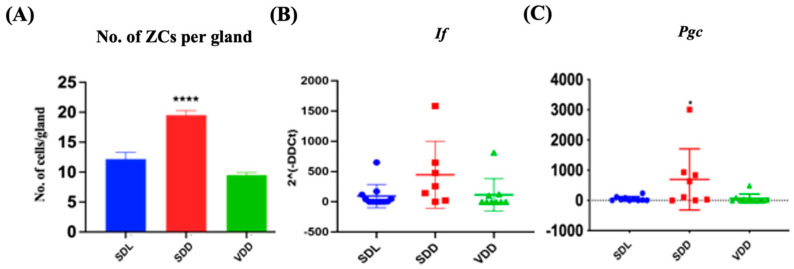
Alteration in Zymogenic Cells in 7M Vitamin D-deficient Mice. (**A**) Quantitative analysis of the zymogenic cell number. Gene expression (2^(-DDCt)) studies for (**B**) *If* and (**C**) *Pgc* in the three groups of mice (*n* = 8–9 per group). Data are presented as the mean ± SE. The one-way analysis of variance was performed for data analysis. * indicates significant differences from the control group. * *p* < 0.05, **** *p* ≤ 0.0001.

H&E staining revealed that the SDD animals had more ZCs in their gastric glands (Figure 3A) than the SDL animals. ZCs develop from mucous neck cells by transdifferentiation and express pepsinogen C (PGC) and intrinsic factor (IF) [12]. Quantifications of the number of ZCs (Figure 4A) and the relative levels of *Pgc* and *If* gene expression (Figure 4B,C) indicated a considerable increase in ZCs in the SDD group.

### 3.4. Parietal Cells Were Overstimulated in Both SDD and VDD Mice

**Figure 5:**
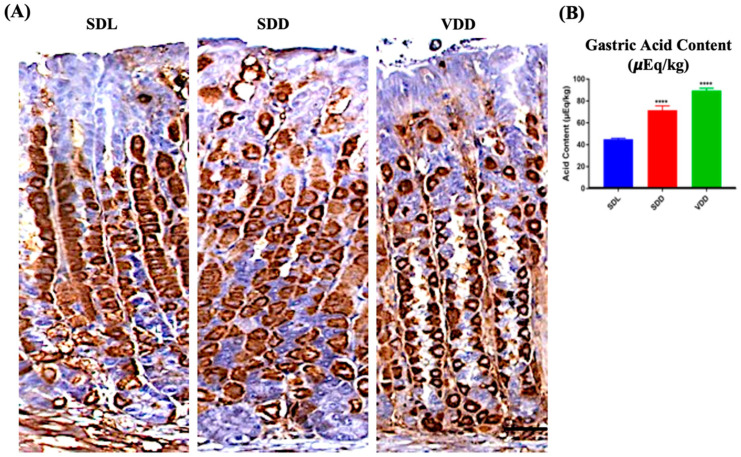
Localization of H,K-ATPase and Measurement of Acid Output in Standard Diet Light (SDL), Standard Diet Dark (SDD), and VD deficient Dark (VDD) Mice. (**A**) Immunohistochemistry for H,K-ATPase (scale bar = 200 μm). (**B**) Gastric acid content measurement (*n* = 7–9 per group). Data are presented as the mean ± SE. The one-way analysis of variance was performed for data analysis. * indicates significant differences from the control group. **** *p* ≤ 0.0001.

Parietal cells secrete gastric acid and express H,K-ATPase protein responsible for the pumping of protons [22]. No major changes were observed between the SDL and SDD groups when the tissue sections were immunoprobed with H,K-ATPase antibodies. The animals in the VDD group, on the other hand, had parietal cells of reduced size. Additionally, out of the 11 animals observed in the VDD group, 7 of them showed a stronger parietal cell staining during immunoprobing using HK-ATPase antibodies (Figure 5A). Remarkably, high acid secretion Figure 5B was observed in these animals despite the parietal cell count and proton pump mRNA expression (Figure S3A,B) showing no major changes. As represented in Figure 5B, the amount of gastric acid increased in the stomachs of the SDD and VDD mice by 72% ± 8.3 μEq/kg (*p* ≤ 0.0001) and 92% ± 4.6 μEq/kg (*p* ≤ 0.0001), respectively.

### 3.5. Upregulation of Muc5ac in the SDD Mice and Downregulation of Muc6 in the SDD and VDD Mice

**Figure 6:**
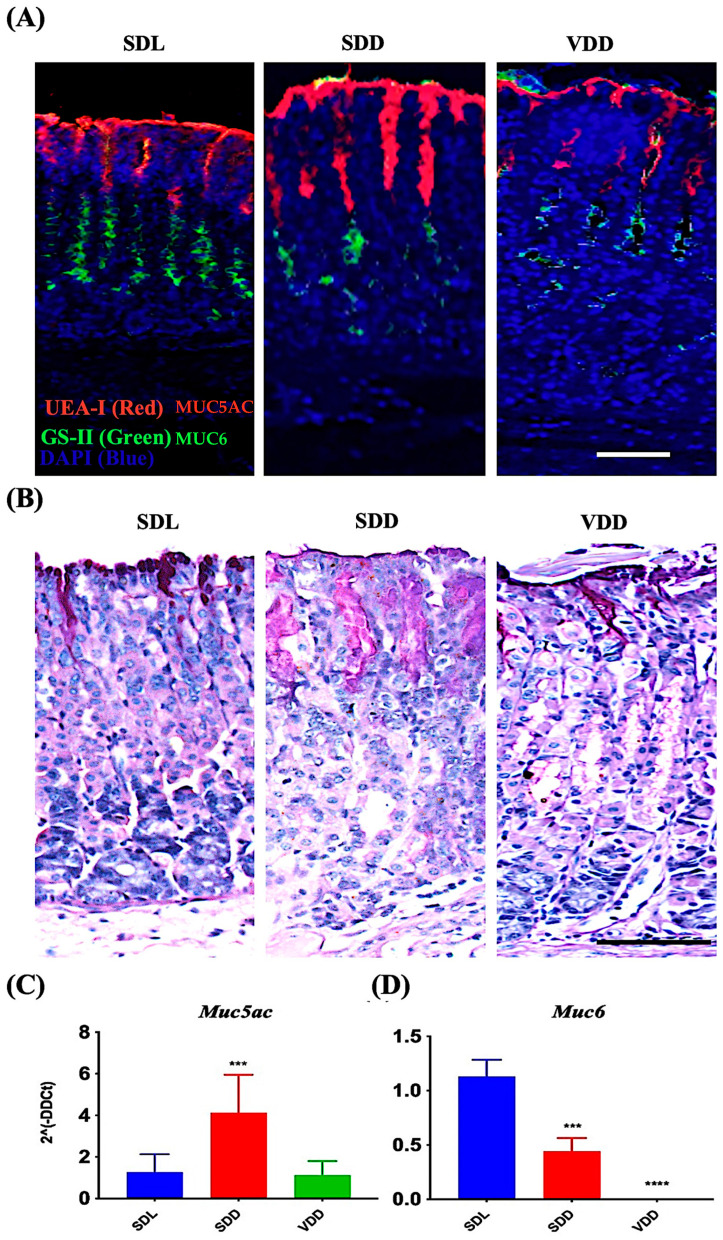
Decreased levels of VD Resulted in Increased Surface Mucous Cells and Reduction in Mucous Neck Cell Lineages. (**A**) Fluorescence labeling with UEA-I and GS-II lectins and (**B**) AB-PAS staining of the stomach mucosa (scale bar = 200 μm). Data of qRT-PCR gene expression study of (**C**) *Muc5ac* and (**D**) *Muc6* (*n* = 8–9 per group) are presented as the mean ± SE and one-way analysis of variance was performed. * indicates significant differences from the control group. *** *p* ≤ 0.001, **** *p* ≤ 0.0001.

MUC5AC and MUC6 are mucin proteins present in the secretory granules of the surface mucus and mucous neck cells, respectively [20,21,22,23,24,25]. Carbohydrate residues, fucose and N-acetyl-D-glucosamine associated with MUC5AC and MUC6, respectively, were identified in surface mucous and mucous neck cells of all mice groups by using UEA-I and GS-II lectins (Figure 6A). Surface mucous cells were identified in the SDL, SDD, and VDD mice by PAS staining method (Figure 6B). Note the increased PAS-labeling in SDD mice as compared to SDL and VDD mice. Expression studies of mucin genes revealed upregulated *Muc5ac* expression in the SDD mice (Figure 6C). However, *Muc6* (Figure 6D) was significantly downregulated in the stomachs of the SDD and VDD mice. *Muc6* expression disappeared in the corpus of the VDD mice.

### 3.6. Mild and Severe Reduction of VD Levels Altered Endocrine Cell Gene Expression

**Figure 7:**
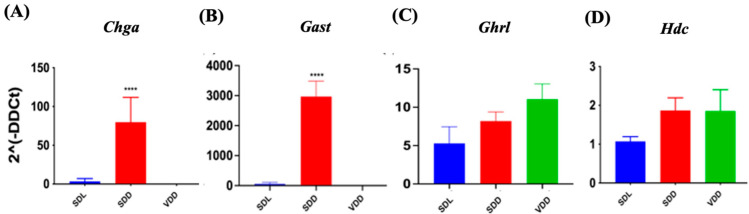
Changes in Gene Expression in Endocrine Cell Lineage Following 7-Month of Vitamin D Deficiency. (**A**) *Chga*, (**B**) *Gast*, (**C**) *Ghrl*, and (**D**) *Hdc* expressions were determined using real-time PCR in the three groups of mice (*n* = 6–9 mice per group). The results are presented as the mean ± SE and significance of changes were determined using one-way analysis of variance. * indicates significant differences from the control group. **** *p* ≤ 0.0001.

Histamine, gastrin, ghrelin, and other factors regulate and maintain acid production at optimum levels in different physiological conditions. To determine how VD deficiency altered acid production in mice stomachs, we evaluated gene expressions of multiple genes (Figure 7). In SDD, the expressions of chromogranin A (*Chga*) (Figure 7A) and gastrin (*Gast*) (Figure 7B) were upregulated by 80% (*p* < 0.0001) and 3000 folds (*p* < 0.0001), respectively. The expression of the ghrelin (*Ghrl*) hormone gene did not vary significantly across the three groups (Figure 7C). Histidine decarboxylase (*Hdc*) (Figure 7D) and gastrin (*Gast*), which are associated with acid secretion, showed no considerable changes in the VDD group. Therefore, we concluded that parietal cells may be overstimulated by another pathway, such as the vagal mechanism, leading to increased acid secretion in 7-month VD-deficient mice.

### 3.7. VD Deficiency Decreased Cell Proliferation

**Figure 8:**
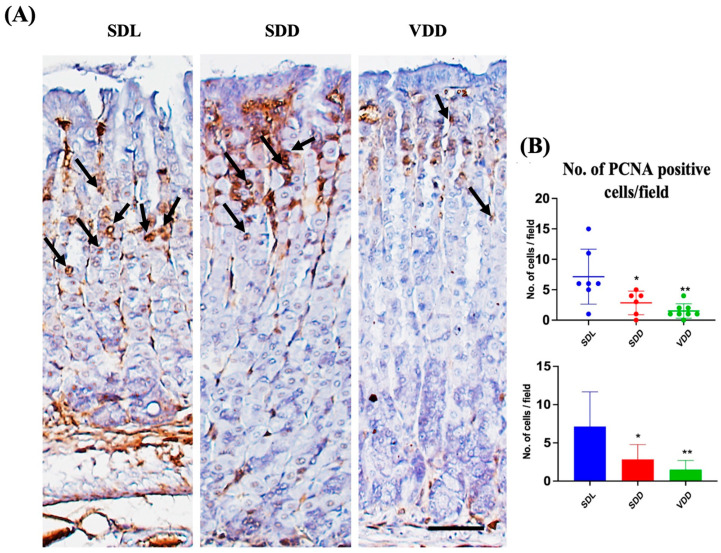
Reduced Proliferation and Increased Apoptosis in Response to Seven-Month Vitamin D Deficiency. (**A**) Assessment of proliferation (black arrows) by the proliferating cell nuclear antigen antibody-based immunohistochemical analysis (scale bar = 200 μm). (**B**) Morphometric analysis of proliferating cells. All mice were seven months old (*n* = 6–9 mice per group). The results are presented as the mean ± SE and the one-way analysis of variance was performed to determine levels of significance. * indicates significant differences from the control group. * *p* < 0.05, ** *p* ≤ 0.01.

To determine the effect of VD deficiency on cellular turnover in the gastric mucosa, cell proliferation and apoptosis were analyzed in the three groups of mice. Antibodies against PCNA were used for immunostaining (Figure 8A) to evaluate cell proliferation. The SDD and VDD mice, as shown by the quantitative analysis in Figure 8B, had significantly fewer PCNA-positive cells than the SDL mice. The analysis of stem cell markers did not reveal any major changes (Figure S4). As shown previously [23], our results imply that decreased cell proliferation in the stomach mucosa are associated with VD deficiency.

### 3.8. VD Deficiency Resulted in the Alteration of the Expression of VDR Target Genes

**Figure 9:**
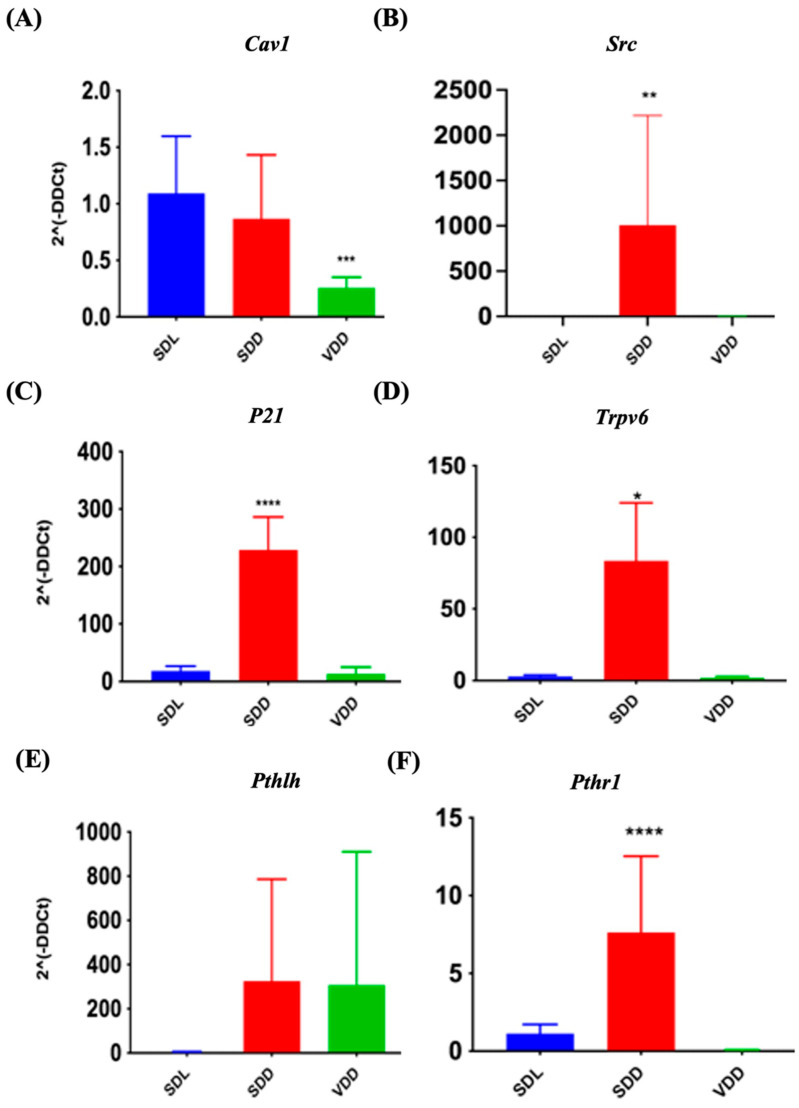
Quantitative Reverse Transcription Polymerase Chain Reaction Validation of protein disulfide-isomerase A3 (PDIA3) and Vitamin D Receptor (VDR) Target Gene Expression. Gene expression studies indicate variations in the expression of PDIA3 target genes (**A**) *Cav1*, (**B**) *Src* and VDR target genes (**C**) *p21*, (**D**) *Trpv6*, (**E**) *Pthlh*, and (**F**) *Pth1r* in VD-deficient mice. All mice were seven months old (*n* = 6–9 mice per group). The results are presented as the mean ± SE. The one-way analysis of variance was performed for data analysis. * indicates significant differences compared with the control group. * indicates significant differences from the control group. * *p* < 0.05, ** *p* ≤ 0.01, *** *p* ≤ 0.001, **** *p* ≤ 0.0001.

VD exerts its biological effects by interacting with VDR and PDIA3. It functions by upregulating or downregulating the target genes. The expression of target genes *Cav1* and *Src*, (for PDIA3) and *P21*, *Trpv6*, *Pthlh*, and *Pthr1* (specific for VDR) were investigated in the present study. In SDD mice, *Src* (Figure 9B), Cyclin-dependent kinase inhibitor 1A (p21) (Figure 9C), *Trpv6* (Figure 9D), and *Pth1r* (Figure 9F) were transcribed at higher levels. Simultaneously, *Cav1* (Figure 9A) was down regulated by 24.8% in the VDD mice. The *Pthlh* gene did not exhibit any significant changes. Immunofluorescence labeling (Figure S5A) and gene expression analysis (Supplemetary Figure S5B,C) did not reveal major changes. In general, our data indicated that VD deficiency might reduce PDIA3 expression in vivo.

## 4. Discussion

The goal of this study was to investigate how 7-month of constant dark exposure alone or with VD deficient diet would affect the gastric glands of mice. The LC–MS/MS analysis of the mice sera yielded interesting findings (Figure 1 and Table S1). Dark exposure alone affected the VD metabolites and mice in the SDD group showed increased levels of the 3-Epi25OHD epimer [5,8,33]. The origin of the C3-epimer and its effect on the body, as well as its link to darkness, are unclear. Numerous studies have shown correlations between VD and weight gain [28,34] or loss [32]. In the present study, the animals were segregated by sex and checked once a week for seven months. We observed variations in the rates of weight gain and loss throughout this period (Figure 2), which was similar to the published results of 12-month-VD-deficient mice [24].

Ghrelin, the “hunger” hormone, is released by X/A-like enteroendocrine cells in the corpus of the stomach. Ghrelin is involved in several physiological processes including the regulation of gastric acid secretion [35], obesity [36], growth hormone secretion [37], and gut motility [38]. We examined ghrelin gene expression to determine whether ghrelin is affected in VDD mice and contributes to weight gain. VD-deficient animals were obese; however, unlike in the 12-month-VD-deficient studies, ghrelin levels did not change considerably. The increased body weight in the SDD mice correlated with the increased adiponectin levels (Figure 2). Interestingly, the 12M group also showed a similar pattern at the same point of time.

In VDD mice, the function of parietal cell lineage is altered. Acetylcholine, gastrin, histamine, and somatostatin regulate acid secretion [23,39]. Compared with the control mice, acid production was higher in the SDD and VDD animals in the present study. As indicated by the gene expression analysis, *Gast* expression was higher by about 3000-fold in the SDD animals, which might be causing the excessive acid secretion. Despite no significant change in *Gast* expression in the stomach of the VDD mice, there was a significant increase in acid output. Neither *Hdc* expression nor the counts of parietal cells reveal any alterations. Increased acid may result from excessive cholinergic stimulation or somatostatin repression in the absence of VD. However, further research is required to confirm these findings. Our data imply that a lack of light and a VD-deficient diet may alter the gene expression of gastrin and perhaps other factor that contribute to increased acid secretion. Unlike the low acid secretion in the 12-month VDD mice, it was interesting to find that the 7-month VD deficiency resulted in higher acid secretion in the VD-deficient SDD and VDD animals. It would be interesting to use TEM to examine any ultrastructural changes in parietal and other cell lineages. Investigating the expression pattern of cellular proteins, such as HIP1R, which are involved in the dynamics of tubulovesicles and secretion of acid by parietal cells [40] may explain the switch from the differential changes in acid secretion with the duration of VD deficiency in mice.

Mucus-secreting cells were altered in the VD-deficient mice. *Muc5ac* expression was upregulated in mice with SDD, whereas *Muc6* expression was downregulated. As mucous neck cells migrate downward toward the base region, they undergo transdifferentiation into ZCs [12,41]. In the 7-month SDD mice ZC numbers increased with increasing *Pgc* expression. This trend was like that seen in the 12M VD-deficient animals. Therefore, it can be concluded that ZC increase starts in the early phase of VD deficiency.

The SDD animals showed upregulated *Gast* expression, resulting in increased stomach acid production and perhaps promoting ZC differentiation [21]. *Muc5ac* and *Muc6* expressions were downregulated in the 7-month VDD mice, whereas, in the 12M VD-deficient animals, the gene expression levels of *Muc5ac* were downregulated in SDD group, and that of *Muc6* was upregulated in the 12M VDD group. However, in the 7M VD-deficient animals, this pattern was altered. To fully understand this phenomenon, further studies are necessary. MIST1, a transcription factor involved in the transition between mucous neck cells and ZCs [42], may help elucidate the mechanism of transdifferentiation and the change in the zymogenic cell numbers in SDD mice.

Spasmolytic polypeptide-expressing metaplasia (SPEM) results from increased number of in mucous neck-like cells expressing TFF2, MUC6, and gastrokine 3 [43,44]. It has been linked to chronic *H*. *pylori* infection and may lead to epithelial carcinogenesis. This condition evolves in response to parietal cell injuries and gastric inflammation. This build-up of transition cells that leads to SPEM may be caused by slowing the development of mucous neck cells into ZCs or dedifferentiation of zymogenic cells [45]. As a result, a few ZC markers were co-labeled with mucous neck cell markers, similar to transition cells or pre-zymogenic cells. SPEM is sometimes referred to as pseudopyloric metaplasia or anterilization [46]. It will be interesting to measure vitamin D levels in a mouse model of SPEM and to test whether vitamin D metabolites are altered. Therefore, signaling pathways of VD might become a target for the prevention and/or treatment of SPEM.

In our previous study, long-term VD deficiency was found to be associated with reduced proliferation [8]. The immunohistochemical labeling of the 7M VD-deficient mice for proliferation revealed comparable effects. A decreased cell proliferation was associated with VD deficiency, suggesting a role for VD in gastric epithelial stem cell proliferation and possibly affect the number of progenitor cells.

The effects of VD are modulated by VDRs and PDIA3 [47]. PDIA3 may regulate proliferation in several cell types [48,49]. In contrast to long-term VD deficiency [9], 7M VD deficiency resulted in the majority of target genes exhibiting substantial changes. Gene expression of *Pdia3* was downregulated in VDD mice stomachs which might have affected cell proliferation in these mice. VDRs and PDIA3 are involved in VD-induced signaling mechanisms [50]. Several concordant upregulated targets, including p21, Trpv6, and Pth1r, were identified by comparing our results with those of previous studies on the effects of long-term VD deprivation on the murine stomach [9]. Caveolin interacts with VDRs and PDIA3 [51]. CAV1 transcription may have decreased in the VDD mice because of PDIA3 suppression. 7M VD deficiency led to the positive regulation of *Src*, *P21*, *Trpv6*, and *Pthr1* in the SDD animals. Because SDD mice contain significant quantities of epimers, these epimers are likely responsible for the increased transcription of these target genes. Further research is necessary to confirm this finding. Unexpectedly, most targets (67%) were upregulated rather than downregulated during VD deficiency caused by a lack of light, indicating the involvement of the epimer as an activator of VDRs and PDIA3 in the stomach mucosa.

Compared to the diet, light has a far greater effect on VD levels and, hence, on stomach homeostasis when discussing its effect. We investigated the gene expression of the melatonin hormone receptors, *Mt1* and *Mt2* (Figure S6) since the SDD and VDD animals were kept in the dark throughout the investigation. Interestingly, in contrast to the mice subjected to chronic VD deficiency, they did not show significant changes. This may be due to the correlation between melatonin and VD levels shown by the pronounced phenotypes observed in the SDD mice. The association between VD and melatonin and the mechanism by which they contribute to the well-being of an organism are not fully understood. These findings suggest a connection between VD and the regulation of tryptophan dehydroxylase (TRPH) gene expression. TRPH is the primary enzyme involved in serotonin synthesis and plays a role in melatonin production [52]. It is widely accepted that insufficient sunlight exposure increases melatonin levels. This results in the inactivation of melanopsin, a photopigment that inhibits melatonin production. Darkness increases the amount of inactive melanopsin, which stimulates the pineal gland to boost melatonin production [52,53]. However, further studies are required to validate these hypotheses.

This study is subject to several limitations that warrant further investigation. Firstly, there is a need to delve into the ultrastructural changes within the gastric stem/progenitor cells and explore the underlying mechanisms responsible for the observed alterations in various gastric epithelial cell lineages. Additionally, it is essential to consider a group of animals exposed to a VD deficient diet under normal lighting conditions. This comparison would not only validate our findings but also distinguish between the effects of darkness and VD deficiency alone, thus introducing an additional dimension to the research. Furthermore, the inclusion of transcriptomic analysis would be a valuable enhancement to our study. Such an analysis could offer a comprehensive view of actively expressed genes and transcripts under varying conditions of VD deficiency, providing a deeper understanding of the molecular mechanisms at play. Using mice as models to study human diseases has its limitations, and one aspect that deserves attention is their nocturnal behavior. When studying the impact of VD deficiency in mice, it is crucial to recognize that while they may be technically VD deficient, their abnormal activity patterns may not adequately replicate the conditions humans experience. This incongruity raises questions about the suitability of mice as a model for studying VD deficiency and highlights the need to explore alternative models that closely resemble the physiological response to VD deficiency observed in humans. In doing so, we can gain a more accurate understanding of the effects of VD deficiency and its potential implications for human health.

## 5. Conclusions

Significant physiological changes occur in animal sera and stomachs during intermediate VD deficiency periods because of a VD-deficient diet and/or darkness. Some phenotypes such as increased epimer levels, ZCs, and apoptosis, as well as reduced cell proliferation, manifest VD deficiency early during the course. However, characteristics such as *Gast* upregulation in SDD, an increase in acid secretion, and mucous cell alteration in both VD-deficient groups may be the result of the body’s attempt to counteract the adverse effects of VD deficiency. This shows that VD is essential for regulating the cellular makeup and acid secretion mechanism of the gastric mucosa. Our results underline the importance of VD obtained from the skin compared with VD obtained from the diet.

## Data Availability

The raw data used to generate the graphs are available upon request.

## Supplementary Materials

The following supporting information can be downloaded at: https://www.mdpi.com/article/10.3390/nu15214648/s1, Figure S1: Flowchart of the Study Design, Figure S2: Levels of serum VD metabolites and VD in hair samples of 7M mice as revealed by LC-MS-MS, Table S1: Representative table showing the different VD metabolites (ng/mL) detected by LC-MS/MS in serum of the three groups of 7M mice, Figure S3: Morphometric and Gene Expression Analyses of Parietal Cells, Figure S4: Gene expression of stem cell markers, Figure S5: VDR and PDIA3 Expression in the Mouse Corpus of 7-month-old Mice, Figure S6: Gene expression of stem cell markers.
