# Supplementary material for: Seven-Month Vitamin D Deficiency Inhibits Gastric Epithelial Cell Proliferation, Stimulates Acid Secretion, and Differentially Alters Cell Lineages in the Gastric Glands
Source: Nutrients. 2023 Nov 2;15(21):4648. doi: 10.3390/nu15214648 (PMC10649607; doi:10.3390/nu15214648)
Supplement: Supplementary file 1 [file nutrients-15-04648-s001.zip › nutrients-2650558-supplementary.pdf]

Supplementary Material

Seven-Month Vitamin D Deficiency Inhibits Gastric Epithelial Cell Proliferation, Stimulates Acid Secretion, and Differentially Alters Cell Lineages in the Gastric Glands

Shaima Sirajudeen [1], Iltaf Shah [2,3], Sherif M. Karam [2,4] and Asma Al Menhali [1,2,*]

1 Department of Biology, College of Science, United Arab Emirates University (UAEU), Al Ain, 15551, United Arab Emirates; 201890080@uaeu.ac.ae
2 Zayed bin Sultan Al Nahyan Center for Health Sciences, United Arab Emirates University (UAEU), Al Ain, 15551, United Arab Emirates; altafshah@uaeu.ac.ae (I.S.); skaram@uaeu.ac.ae (S.M.K.)
3 Department of Chemistry, College of Science, United Arab Emirates University (UAEU), Al Ain, 15551, United Arab Emirates
4 Department of Anatomy, College of Medicine and Health Sciences, United Arab Emirates University (UAEU), Al Ain, 15551, United Arab Emirates
* Correspondence: asmaa@uaeu.ac.ae

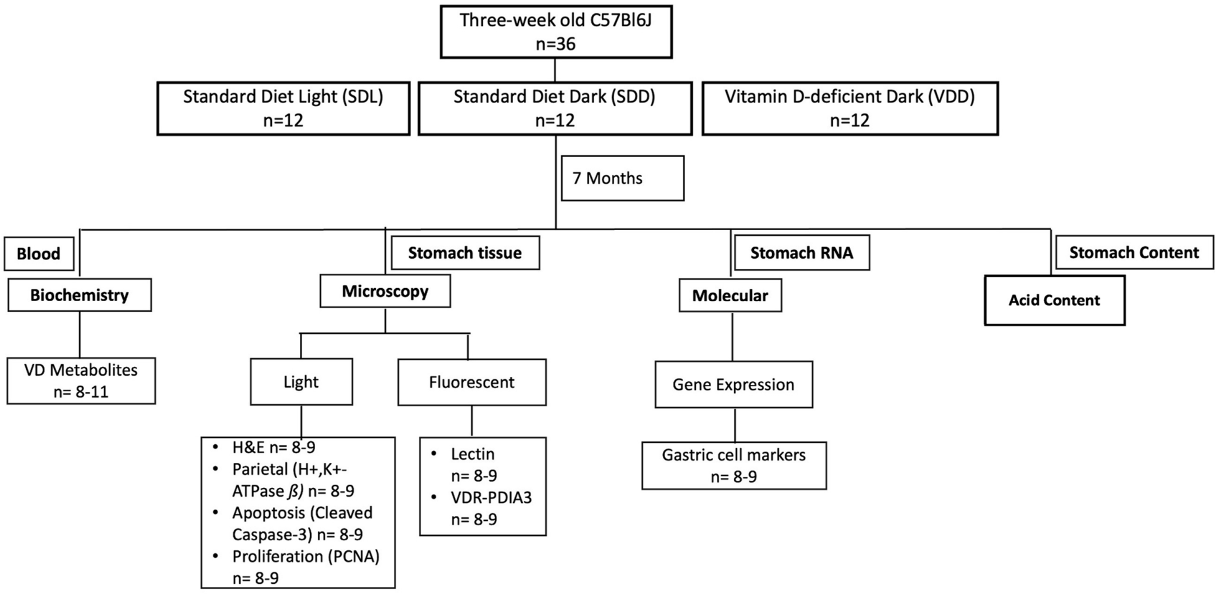

Figure S1. Flowchart of the Study Design

C57Bl6J mice (n=36) were divided into 3 groups – SDL (n=12), SDD (n=12) and VDD (n=12) for seven months according to the type of diet fed and lighting conditions. At the end of the experimental period, the mice were sacrificed, and the samples were collected for different experiments.

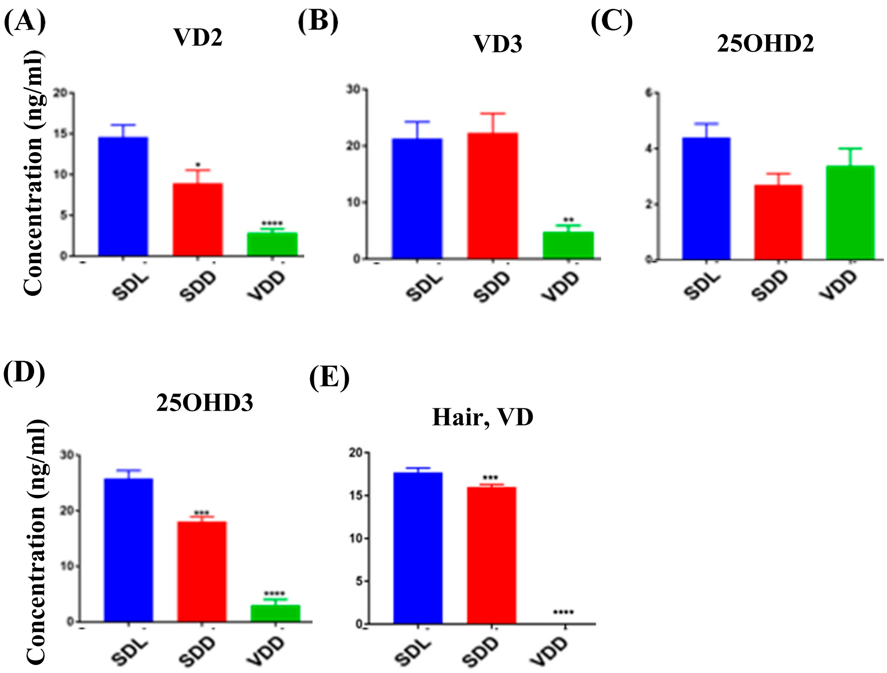

Figure S2: Levels of serum VD metabolites and VD in hair samples of 7M mice as revealed by LC-MS-MS

All mice were 7 months old, n = 8–11 per group. Data are presented as mean ± SE. One-way ANOVA was used for data analysis. * indicates significant differences from the control group. * $p < 0.05$, ** $p \leq 0.01$, *** $p \leq 0.001$, **** $p \leq 0.0001$.

Table S1: **Representative table showing the different VD metabolites (ng/ml) detected by LC-MS/MS in serum of the three groups of 7M mice.**

SDL

Sample Name	Serum volume (µl)	VD3	VD2	VD	25OHD3	25OHD2	25OHD	3-Epi25OHD3
**SDL3**	25.0							
**SDL5**	120.0	25.2	12.0	37.2	29.0	5.1	34.1	3.5
**SDL7**	83.0	12.8	15.0	27.8	27.0	3.3	30.3	2.8
**SDL8**	95.0	18.2	12.0	30.2	21.0	3.1	24.1	3.1
**SDL9**	240.0	30.0	14.0	44.0	25.0	5.2	30.2	5.3
**SDL10**	160.0	20.4	20.0	40.4	27.5	5.3	32.8	3.3
**Average**		**21.3±6.6**	**14.6±3.3**	**35.9±6.8**	**25.9±3.1**	**4.4±1.1**	**30.3±3.8**	**3.6±1**

SDD

Sample Name	Serum volume (µl)	VD3	VD2	VD	25OHD3	25OHD2	25OHD	3-EPI25OHD3
**SDD1**	135.0	18.2	12.5	30.7	20.0	2.2	22.2	7.2
**SDD2**	128.0	16.8	8.7	25.5	16.5	3.5	20.0	6.5
**SDD3**	160.0	32.0	8.0	40.0	18.0		18.0	6.4
**SDD4**	80.0		3.5	3.5	16.0	2.4	18.4	6.0
**SDD5**	50.0							
**SDD8**	180.0	22.2	11.9	34.1	20.0		20.0	8.5
**Average**		**22.3±6.9**	**8.9±3.6**	**26.8±14**	**18.1±1.9**	**2.7±0.7**	**19.7±1.7**	**6.9±1**

VDD

Sample Name	Serum volume (μl)	VD3	VD2	VD	25OHD3	25OHD2	25OHD	3-EPI25OHD3
VDD3	80	6.4	2.2	8.6	0.5	5.0	5.5	
VDD6	120		2.3	2.3	0.6		0.6	
VDD7	220	3.4	4.0	7.4	1.8		1.8	1.4
VDD9	180	7.0		7.0	4.6	2.0	6.6	
VDD10	260	2.4		2.4	7.5	3.5	11.0	
VDD11	110		3.9	3.9	4.8	3.0	7.8	
VDD12	80		2.0	2.0	1.7		1.7	1.7
Average		**4.8±2.2**	**2.9±1.0**	**4.8±2.8**	**3.1±2.6**	**3.4±1.3**	**5.0±3.8**	**1.6±0.2**

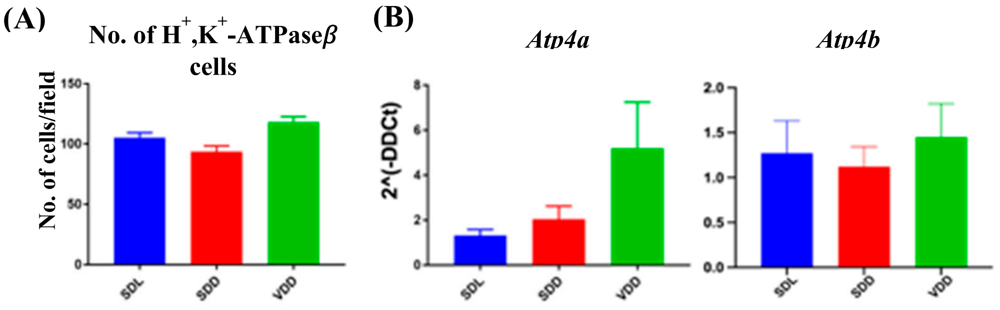

Figure S3: Morphometric and Gene Expression Analyses of Parietal Cells

(A) Quantitative analysis of H+,K+-ATPase ß-labeled cells (five glands per field). (B) Gene expression analyses for Atp4a and Atp4b. All mice were 7 months old, n = 8-9, per group and statistics are presented as mean ±SE. One-way ANOVA was used for data analysis.

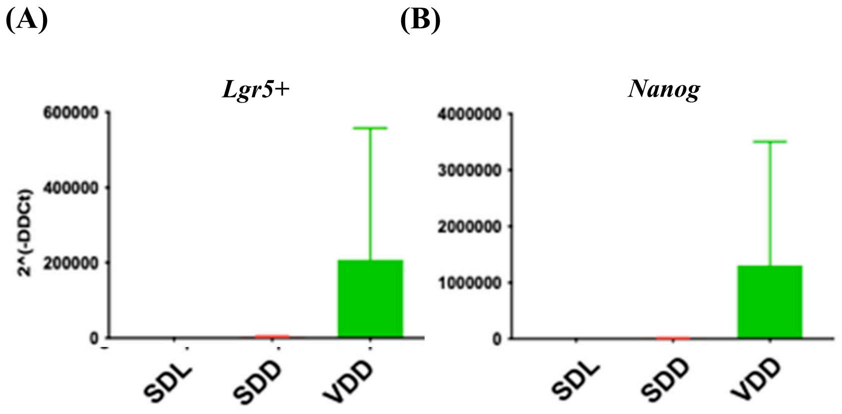

Figure S4: Gene expression of stem cell markers

Gene expression studies for **(A)** *Lgr5+* and **(B)** *Nanog* in the stomach corpus. All mice were 7 months old, n = 8–9 per group. Data are presented as mean ± SE. One-way ANOVA was used for data analysis.

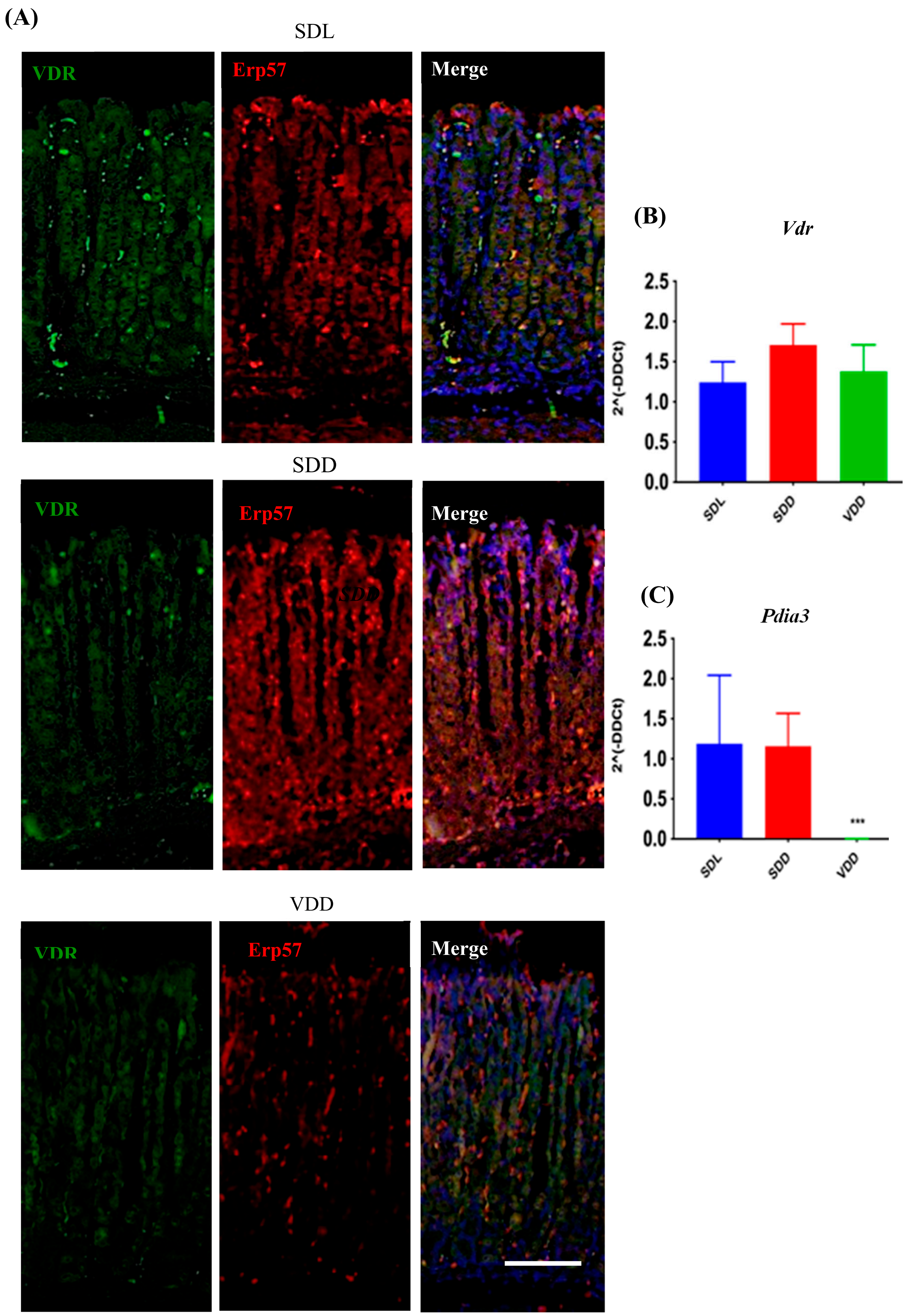
(A)
SDL
VDR
Erp57
Merge
SDD
VDR
Erp57
Merge
VDD
VDR
Erp57
Merge
(B)
Vdr
2^(-DDCt)
2.5
2.0
1.5
1.0
0.5
0.0
SDL
SDD
VDD
(C)
Pdia3
2^(-DDCt)
2.5
2.0
1.5
1.0
0.5
0.0
***
SDL
SDD
VDD

Figure S5: VDR and PDIA3 Expression in the Mouse Corpus of 7-month-old Mice

(A) Anti-nuclear VDR and anti-Erp57 (PDIA3) antibodies were used in the IHC investigation for the three groups of mice (scale bar = 500 µm). **(B)** *Vdr* and **(C)** *Pdia3* expression levels were determined by qRT-PCR. All mice were 7 months old, n = 6–9 per group. The results are presented as mean ±SE. One-way ANOVA was used for data analysis. * indicates significant differences from the control group. *** $p \leq 0.001$.

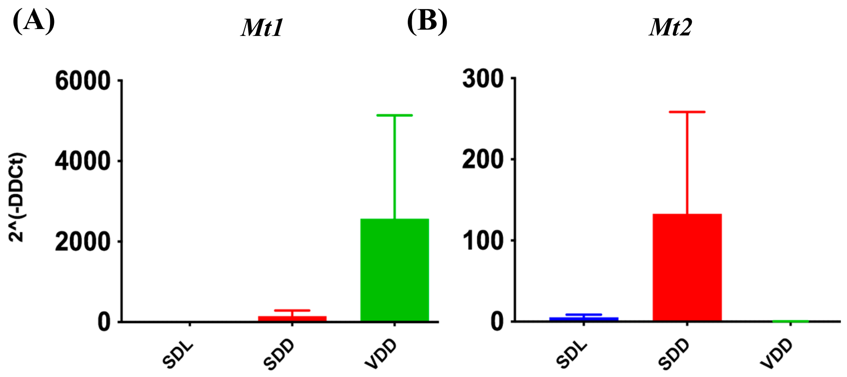

Figure S6: Gene expression of melatonin receptors

Gene expression studies for **(A)** *Mt1* and **(B)** *Mt2* in the stomach corpus. All mice were 7 months old, n = 8–9 per group. Data are presented as mean ± SE. One-way ANOVA was used for data analysis.
